# Okapi-EM: A napari plugin for processing and analyzing cryogenic serial focused ion beam/scanning electron microscopy images

**DOI:** 10.1017/S2633903X23000119

**Published:** 2023-03-27

**Authors:** Luís M. A. Perdigão, Elaine M. L. Ho, Zhiyuan C. Cheng, Neville B.-Y. Yee, Thomas Glen, Liang Wu, Michael Grange, Maud Dumoux, Mark Basham, Michele C. Darrow

**Affiliations:** 1 Artificial Intelligence and Informatics, The Rosalind Franklin Institute, Didcot, UK; 2 School of Chemistry, University of Edinburgh, Edinburgh, UK; 3 Structural Biology, The Rosalind Franklin Institute, Didcot, UK; 4 Division of Structural Biology, Wellcome Centre for Human Genetics, University of Oxford, Oxford, UK; 5 Diamond Light Source, Didcot, UK

**Keywords:** Cryogenic, image processing, napari, serial FIB/SEM, software, 3D imaging

## Abstract

An emergent volume electron microscopy technique called cryogenic serial plasma focused ion beam milling scanning electron microscopy (pFIB/SEM) can decipher complex biological structures by building a three-dimensional picture of biological samples at mesoscale resolution. This is achieved by collecting consecutive SEM images after successive rounds of FIB milling that expose a new surface after each milling step. Due to instrumental limitations, some image processing is necessary before 3D visualization and analysis of the data is possible. SEM images are affected by noise, drift, and charging effects, that can make precise 3D reconstruction of biological features difficult. This article presents Okapi-EM, an open-source napari plugin developed to process and analyze cryogenic serial pFIB/SEM images. Okapi-EM enables automated image registration of slices, evaluation of image quality metrics specific to pFIB-SEM imaging, and mitigation of charging artifacts. Implementation of Okapi-EM within the napari framework ensures that the tools are both user- and developer-friendly, through provision of a graphical user interface and access to Python programming.

## Impact Statement

Cryogenic serial plasma focused ion beam milling scanning electron microscopy is an emerging microscopy technique that is used to visualize 3D structures of biological features at mesoscale resolutions. This technique requires common postprocessing of data such as alignment and charge mitigation to enable robust segmentation and analysis. In addition, approaches are needed to quantify data quality to enable an assessment of features and tune data acquisition parameters to enable optimal image acquisition. This article presents Okapi-EM, a combination of software tools designed to facilitate these important initial steps in assessing and processing images from these experiments. These tools have been assembled as a plugin for a popular 3D biological image visualizer called napari, making their usage user-friendly and readily accessible.

## 1. Introduction

Recent advances in cryo-electron microscopy hardware have seen an emergence of instruments which combine microscopy techniques and milling instruments within the same system. Dual-beam focused ion beam scanning electron microscopy (FIB/SEM), maintained at cryogenic temperatures, provides a workflow to acquire volumetric SEM images of a range of biological samples in their near-native state at nanometer-resolution^(^^1^^–^^3^^)^. This technique builds volumetric representation of the specimen by cyclic FIB-milling (to remove the freshly imaged surface) and SEM imaging of the specimen. Several SEM images (typically hundreds) are obtained, corresponding to decreasing heights of the specimen, which can be computationally stacked to obtain a volumetric representation of the sample being measured.

Serial FIB/SEM has historically been used to capture images of fixed, stained, and resin-embedded samples providing cellular and subcellular imaging in 3D that can be used for the reconstruction and analysis of biological features. However, fixation, staining, dehydration, and resin embedding can introduce artifacts^(^^4^^–^^8^^)^. With the development of cryogenically stable microscope stages and reduced rates of ice formation, it is possible to image cryogenically prepared samples providing structural information of these samples in near-native states. Cryogenic serial pFIB/SEM has been done using modified room temperature plasma ion beam milling microscopes which were designed for physical science applications and more recently has been further enabled through the development of fit-for-purpose commercial options, some of which offer multiple plasma generation gases^(^^1^^,^^9^^–^^14^^)^.

As with many experimental techniques which produce 3D data, it is often desirable to annotate biological features and to visualize the structure in three dimensions (3D), but important preprocessing steps are needed before data is suitable as input for these segmentation tasks. For instance, small translational movements between images within the stack caused by stage and/or sample movement are often observed as misalignment between images; while compensatory functions exist within the instrument it is not always possible to correct on-the-fly. Therefore, these must be compensated for, otherwise volumetric segmentation^(^^15^^,^^16^^)^ and computational counting tools like connected components algorithms will fail or struggle to succeed. Additionally, SEM images of biological samples often contain artifacts caused by charging around insulating substances such as lipids^(^^3^^,^^12^^)^. Automatic and semi-automatic segmentation tools require aligned datasets and data that can be effectively normalized to remove any strong features generated by sample charging. Another common issue observed during pFIB/SEM imaging is the creation of curtaining artifacts during the milling step which are then visible as streaks in the milling direction during SEM imaging. Finally, having quantitative tools that assess the quality of the data under certain imaging (e.g., optimal focus and voltage) and milling conditions (e.g., focusing, curtaining, and milling accuracy) will assist in the generation of data that best mitigate these factors, resulting in optimal further processing and optimization of future data acquisition strategies^(^^15^^,^^16^^)^. These necessary presegmentation tasks can be time-consuming and often require use of multiple pieces of software or bespoke code. Okapi-EM provides a selection of tools which address some of these needs in a single software package found within napari^(^^17^^)^. In this first release of Okapi-EM, there are three tools available:

1. Stack Alignment. This tool provides the user with appropriate transformation options for the alignment of stacks of slices.
2. Charge mitigation (Chafer). This tool requires presegmented “charge centers,” then applies filters to mitigate the charging artifacts found nearby.
3. Resolution estimation (Quoll). This tool requires microscope calibration and provides a measure of the mean resolution and standard deviation for individual slices.

Similar collection of tools for processing serial-FIB/SEM data are also available in *bmiptools*^(^^18^^)^ software package, which was developed independently of Okapi-EM. However, this is not a napari plugin, therefore requiring users knowledge of programming in order to use it. Unfortunately, at the time of writing this article (bmiptools version 1.0.1), execution of its charge artifact removing filter was attempted in our image stack data set (see below), but it resulted in no difference and no suppression.

Example data used throughout this manuscript are available on EMPIAR (see Data Availability) and their sample preparation and pFIB/SEM data collection have been described in Dumoux *et al.*^(^^1^^)^ and are briefly detailed here. Biological samples were vitrified by plunge freezing (yeast) or by high-pressure freezing (mouse brain). The microscope used is a dual beam FIB/SEM “Helios G4 Hydra” equipped with an Aquilos II type cryo-stage. SEM electron beam voltage and current were 1 kV and 12.5 pA for the yeast and 1.1 kV and 6.25 pA for the brain. Quoted SEM image angles are relative to sample plane. Argon was used for milling, and the accelerating voltage used was 20 kV.

Typically, plasma-FIB (pFIB) uses ionized gases as milling source, and traditional FIB often refers to ion beam using liquid metal sources, such as gallium. We highlight here that Okapi-EM was designed to process pFIB-SEM images, as it has been thoroughly tested with this data. Nevertheless, Okapi-EM may prove to be equally useful to process data generated by other ion beam source, though usage of Okapi-EM with this type of data is untested.

## 2. Implementation

Napari is an open-source, user-friendly, data visualization application that runs in Python programming language^(^^17^^)^. It supports three-dimensional visualization of different data types (i.e., image and labels) interactively. Its annotation features are useful in biological image analysis and processing. Furthermore, its functionality can be extended with support for plugins, allowing continuous development of image processing methods alongside of experimental microscopy advances. Developing within the Python ecosystem also facilitates deep learning approaches with easy access to modern machine learning packages such as scikit-learn^(^^19^^)^ and PyTorch^(^^20^^)^. The plugin installation uses the well-established package management system (PyPI), which also enables plugin installation chains, where a single plugin can install many other plugins or packages as needed to create a *bundle.* Bundling several plugins in this way means that a whole toolbox can be created for specific data processing workflows or research themes (e.g., devbio-napari^(^^21^^)^, or napari-assistant^(^^22^^)^, both of which were tailored for biological image processing). Napari supports and opens a range of image formats through the *skimage* library, including multi-page tiff. It also hosts a variety of other image processing and file format plugins so Okapi-EM tools can be integrated into and combined with other workflows. Okapi-EM can be installed through napari’s plugin search and installer engine. Otherwise, it can be downloaded freely at https://github.com/rosalindfranklininstitute/okapi-em, or installed from Python package index (PyPI). Okapi-EM was developed for minimum python version 3.7 and installations of other dependency packages using PyPI or napari’s plugin installation engine are automatic.

**Figure 1.**
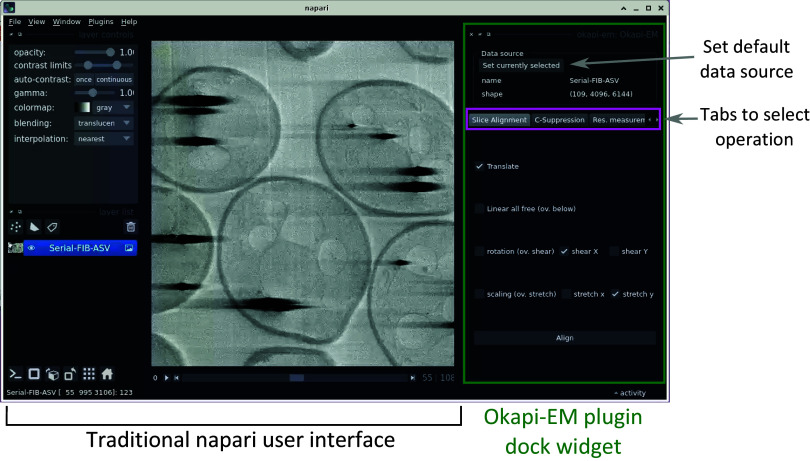
View of the napari application highlighting the Okapi-EM plugin (green rectangle at right), and the currently available tools (pink rectangle at right). Each Okapi-EM plugin has its own tab with appropriate options for its use displayed. See Supplementary Figure S1 for a detailed view and description of the options available in each plugin.

Currently, Okapi-EM incorporates three major image processing tools which are organized in the user interface as separate tabs: stack alignment, charge suppression, and resolution measurement (Figure 1). Each tab contains user interface elements to adjust settings and execute data analysis.

## 3. Stack Alignment

In FIB-SEM, samples are milled in preparation for imaging. During this process, a number of issues can cause drift or misalignment in the image stack, such as the movement of the sample due to mechanical stress, small temperature variations and slight, iterative, stage movements going from milling to imaging positions that accumulate over time^(^^23^^)^. Inaccurate placement of the milling area by the operator or software may also lead to the observation of shearing during subsequent imaging^(^^24^^)^. These misalignments may further be amplified by factors including charging effects and instabilities caused by external disturbance^(^^24^^)^. As a result, an important element in the alignment of the resultant SEM images is the shearing or skewing along the direction perpendicular to the ion beam (or other layer removal option) trajectory^(^^23^^)^, which is particularly significant along the slow scan direction. Before any subsequent visualization or analysis tasks, aligning the image stack is a crucial step.

While there have been efforts at improving image acquisition hardware and developing real-time correction^(^^25^^–^^27^^)^, these methods are limited by the need to ensure that the sample is not overexposed during data collection. Therefore, they do not provide fine alignment correction and require the use of subsequent alignment software^(^^24^^)^. Several software packages/plugins are available for this task, such as the closed-source and pay-for-service Amira by ThermoFisher^(^^28^^)^ and open-source options such as ImageJ plugins Linear Stack Alignment with SIFT^(^^29^^)^ and TrakEM2^(^^30^^)^. Although these software can in general align the images so that there is smooth transition between the slices, a major drawback is that they do not consider the physical process by which the images were acquired, thus they can introduce distortions that are not present in the sample. During alignment, if incorrect transformation parameters are chosen, because slices are aligned to their neighbors, this can cause a cascade of transformations that can ultimately distort the shape of the stack^(^^31^^)^. Notably, the ImageJ plugins Linear Stack Alignment with SIFT and TrakEM2 do not offer options to perform alignment with shearing instead of rotation in particular directions, which is what images obtained by scanning line-by-line need to be compensated for.

### 3.1 Alignment method

We have developed an improved image alignment method that aims to provide users with a variety of transformation options to appropriately describe image acquisition processes and to reduce the likelihood of introducing nonphysical distortions during alignment of 3D stacks. It employs scale-invariant feature transform, or SIFT, a widely used algorithm for the detection of local landmarks^(^^32^^)^. Since SIFT tends to include some false matches that could affect the alignment outcome, filtering is necessary to remove these outliers. Users can choose between percentile and RANSAC filters to achieve this. The former option is faster but less robust, while the latter is more computationally costly, but more reliable in cases where matches are scarce, or exhibiting misalignment. Users will then define a transformation that would best describe the physical distortion of the images depending on the image acquisition technique (Table 1) by adding or removing components of the transformation matrix, including translation, rotation, shearing, uniform scaling and stretching or shrinking, or selecting affine transformation which allows all six degrees of freedom. A description of each option is shown in Supplementary Figure S2. For instance, if the option shearing along the 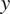 -axis is enabled, and no scaling or shearing in 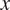 -axis are chosen, then the transformation matrix and translation vector to be obtained is:(1)

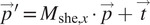

**Table 1.** Types of transformations that are commonly used to correct distortions from different volumetric electron microscopy techniques.

	Translation	Rotation	Shear	Scale	Stretch	Affine
Block-face imaging, laser sectioning (e.g., FIB/SEM, cryo FIB/SEM)	Yes	No	In scanning direction	No	In scanning direction	No
Block-face imaging, mechanical sectioning (e.g., SBF SEM)	Yes	No	In scanning and sectioning directions	No	In scanning direction	No
Imaging of thin serial sections (e.g., ssTEM)^a^	Yes	Yes	In sectioning direction	No	No	Yes
Tomographic imaging of thin serial sections (e.g., electron tomography)^b^	Yes	Yes	In scanning direction	No	No	Yes

a Deformations between subsequent serial section images.

b Deformation between projection images of the same thin section taken at different tilt angles.

or:(2)

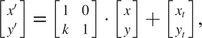

where 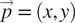 is a feature point on the source image and 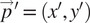 is its location after shearing with 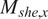 being the linear transform for shearing along 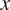 by the parameter of 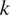 , and translation of 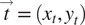 .

The alignment algorithm starts from the first image slice and calculates the optimized transformation parameters between consecutive slices along the whole stack. Similar features between images are identified and matched. The distance between a pair of feature points is 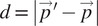 and, considering all the feature points pairs between images, the sum of the squared distances is minimized by adjusting the parameters 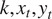 .

### 3.2 Rotational distortion introduced with existing software

**Figure 2.**
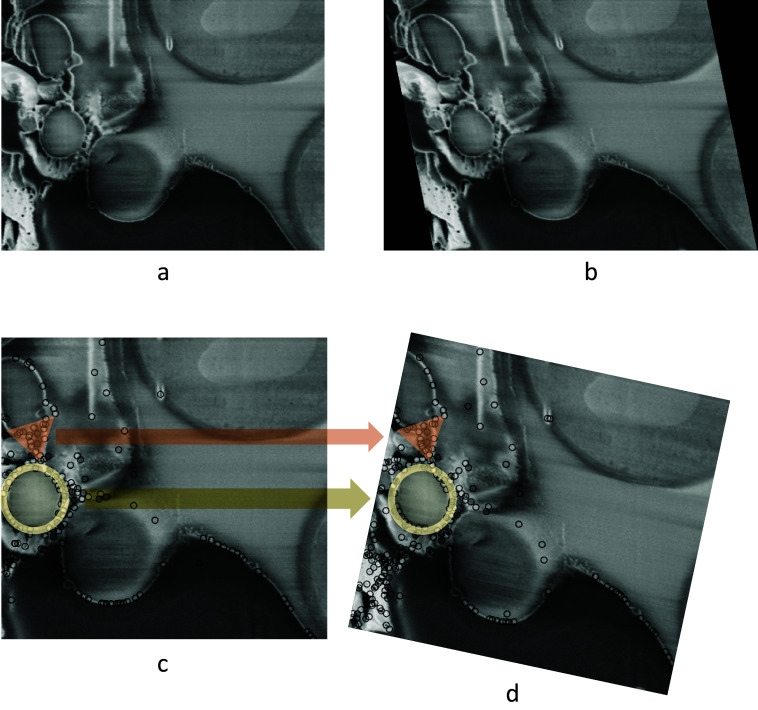
Nonphysical distortion in the alignment process if the modes of transformation are not appropriately limited. (a) Original SEM image of yeast cells. (b) Artificially distorted image after a shearing transformation was applied to the original image. A shear factor of 0.15 was selected for visibility. (c) Feature points found in (b) using SIFT, with two feature-dense regions highlighted with circle and triangle markers. (d) Possible alignment result when the modes of transformation are not restrained, where even though the feature-rich regions are well-aligned, a nonphysical rotation is introduced. Data is FIB-SEM of yeast, available at EMPIAR with ID 11416.

As discussed previously, limiting the modes of transformation according to the physical process of image acquisition is crucial. Failing to do so when aligning FIB-SEM image stacks could result in a large angle rotation in the image slices that is not actually present in the sample. To demonstrate this, an artificial shearing transformation was applied to the original image (Figure 2a) acquired using cryogenic serial pFIB-SEM to mimic a shear force distortion (Figure 2b). For this image pair, using SIFT, many matches can be found on the left side of the image, especially in the triangular and circular region (Figure 2c). Using least square or other optimization methods without restraining the transformation mode results in a large angle rotation or an affine transformation that aligns those feature-rich regions well, but fails to align other parts of the image and introduces a nonphysical distortion (Figure 2d).

### 3.3 Results

**Figure 3.**
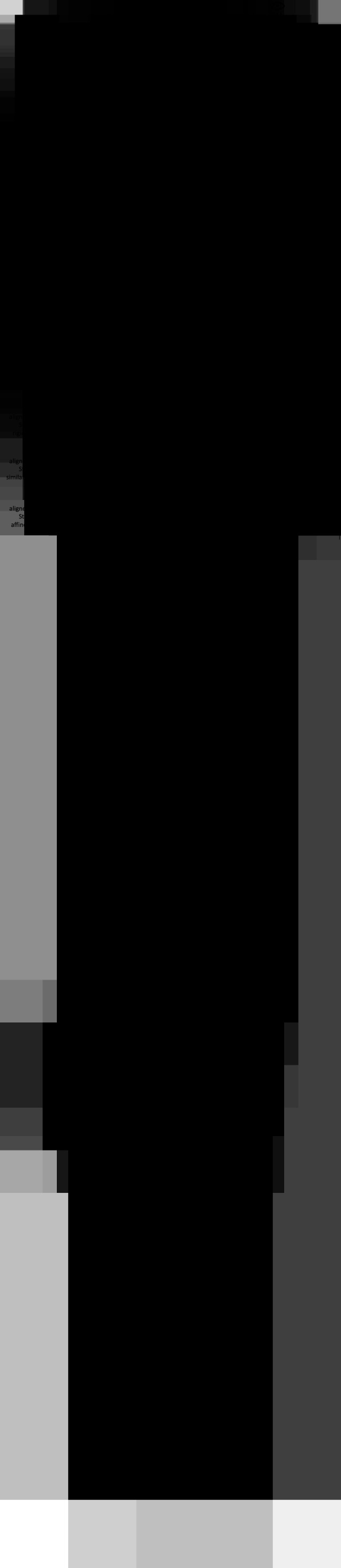
Alignment results and comparison between Okapi-EM, TrakEM2, and Fiji Linear Stack Alignment with SIFT (a) Cross-sectional views of SEM image stack of yeast cells (109 slices along *z* direction, slice size 20.7 × 13.8 μm^2^) are obtained. (b–i) Cross-sectional view of a 11.2 × 3.7 μm^2^ area at *y* = 2 μm of the unaligned stack and the aligned stacks using Okapi-EM alignment with RANSAC and {translate, shear *x*, stretch *y*} selected, Fiji plugin TrakEM2 with rigid transformation, similarity transformation, or affine transformation selected, respectively, and Fiji plugin Linear Stack Alignment with rigid transformation, similarity transformation, or affine transformation selected respectively. (j–q), Cross-sectional view of a 15.0 × 3.7 μm^2^ area at *y* = 11 μm of the aligned stacks using the three aforementioned alignment methods and settings respectively. Data is EMPIAR-11416. In both Fiji plugins, rigid transformation allows translation and rotation. Similarity transformation allows translation, rotation, and scaling. Affine transformation is defined as shown in Supplementary Figure S2. Details about the rendering of the cross-section images is detailed in Supplementary Figure S8.

Okapi-EM stack alignment was applied to a cryogenic serial pFIB/SEM image stack of yeast cells (109 slices). As described in Table 1, translation, shear *x–*axis, and stretch along *y-*axis were chosen to compute an alignment based on the landmarks found using SIFT with RANSAC outlier filtering. Cross-sectional views are used to visualize the alignment result (Figure 3a). To compare outcomes, the same image stack was aligned in Fiji plugins TrakEM2 and Linear Stack Alignment with each transformation option offered as well. Substantial drift and distortion can be observed in the unaligned stack (Figure 3b,j and Supplementary Movie S1). After alignment, cell shapes are restored with mostly smooth outlines (Figure 3c,k and Supplementary Movie S2). While all alignment approaches improved the cross-sectional alignment to some degree, distortion can still be observed in the outline of the cells when using either TrackEM2 or Linear Stack Alignment (Figure 3d–i,l–q), especially at the *y* = 2 μm when using TrakEM2 and when affine transformation is selected in Fiji Linear Stack Alignment.

## 4. Charge Artifact Mitigation

**Figure 4.**
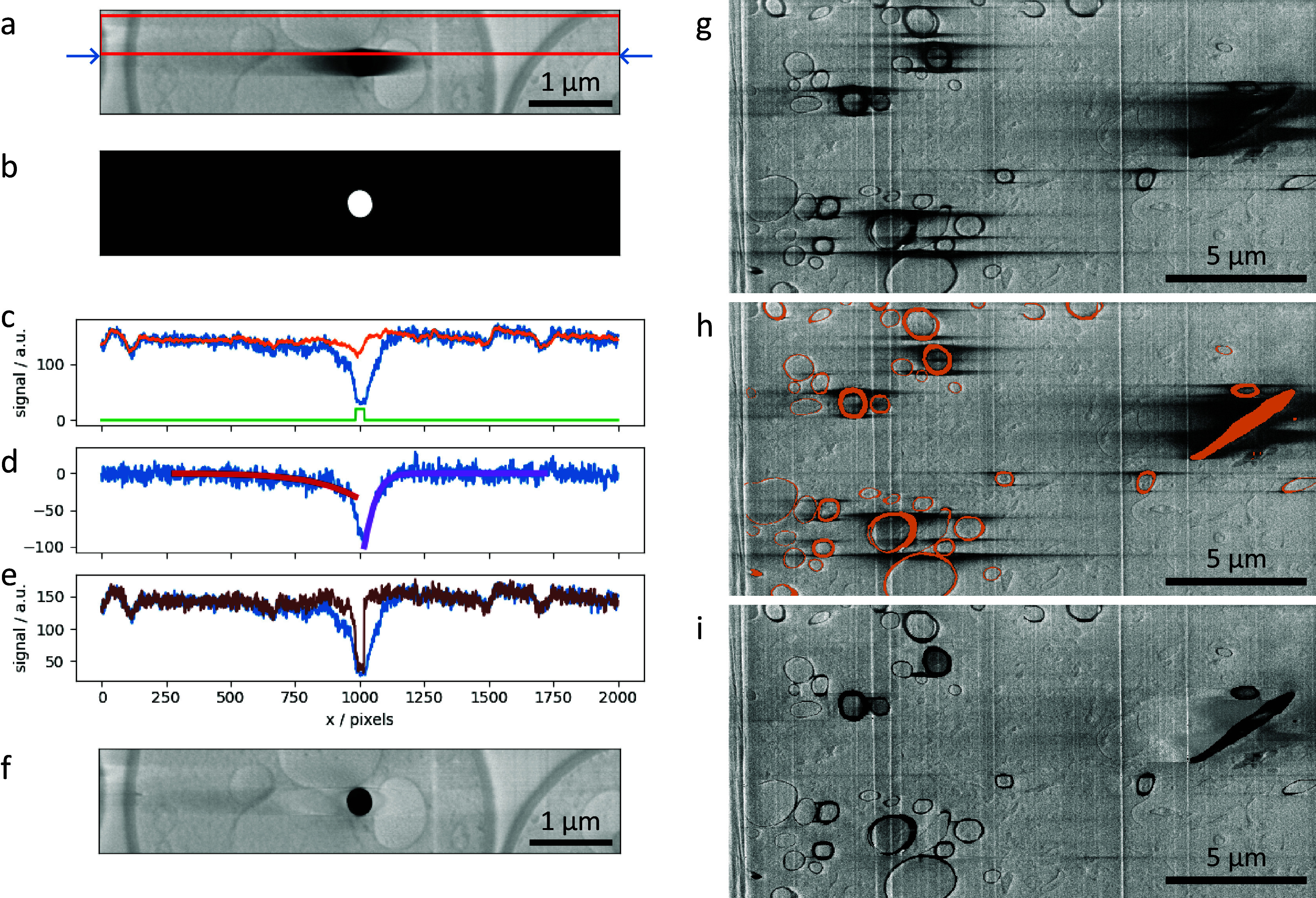
(a–f) SEM images and plots illustrating the charge artifact removal algorithm on a lipid droplet from a yeast dataset. Data is EMPIAR-11416. (a) SEM image of a lipid droplet within a yeast cell (image size 6.75 × 1.35 μm^2^). Arrows indicate a scanning line of interest, with its signal profile in (c–e) as blue line. Red rectangle represents data region where signal was averaged (width set by the nlinesavaerage parameter) resulting in signal profile in (c) as orange line. (b) Annotation image of the charge center, corresponding to the charging artifact in Figure (a). Green line in plot (c) is the line profile of the annotation along the row of interest (arbitrary units). (d) Line profiles describing the background corrected signal (blue line) and the optimized functions 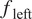 and 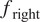 , (red and purple) respectively. (e) Line profiles of the uncorrected signal (blue line) and corrected signal (brown line). (g–i) SEM images illustrating charge artifact suppression on myelinated sheaths found in mouse brain (image size 20.7 × 10.3 μm^2^). Data is EMPIAR-11415. (g) Original SEM image displaying copious amounts of charging. (h) SEM image with overlaid segmentation of charging centers and extending to complete rings of myelin sheaths. (i) Result after applying filter with default parameters.

Charging artifacts often appear when insulating materials interact with the electron beam^(^^1^^,^^3^^)^. This effect is normally minimized by adjusting beam energy or scanning parameters, and its severity depends on the target substance being imaged. Biological samples can present a challenge, with cells often containing different compartments with distinct electrical conduction properties that cannot be completely balanced through adjustments in acquisition parameters. Inevitably, images will show evidence of charging, causing artifacts which manifest in the SEM images collected in the form of elongated dark regions in the scanning direction that extend asymmetrically beyond the charging feature itself (Figure 4a,g). The practical outcome of this artifact is the partial obscuring of biological features nearby to insulating materials. This makes both manual and automated downstream data processing and analysis (e.g., segmentation or visualization) more difficult.

A previous attempt to mitigate charging in SEM images presented by Spehner *et al.*^(^^3^^)^ utilizes the python scikit-image dilation/morphology function^(^^33^^)^. This method separates the charging tail signals from the SEM images and then partially subtracts them from the original image. In our hands, this method did not work with the charging artifact tails in our datasets (Supplementary Figure S6), possibly indicating it is specific to the datasets it was developed for or to the instrument used to collect them.

Okapi-EM contains a charging artifact suppression tool^(^^34^^)^ which is designed to restore the image contrast within and around charging artifacts, while retaining the charge centers themselves. As the artifact appears elongated along the direction of scanning (laterally) and is therefore influenced by the rastering nature of the scanning, it immediately suggests that to reverse this effect in the images, a row-by-row filter that uses information of the surrounding areas, in particular along the direction perpendicular to scanning, should be used to subtract the charging effect. With Okapi-EM’s chafer, the restoration method operates sequentially row-by-row in down and up passes, and the estimation of the charging artifact tails is done by fitting with a smoothing function, which is later subtracted.

For this filter to work a semantic segmentation of the charging centers must be provided as a separate layer in napari. Within the plugin user interface, there is a field where user sets this layer as being the annotation of the charging artifacts (Supplementary Figure S1B). This segmentation can be done manually or by using shallow or deep-learning^(^^35^^)^ predictors using either tools within napari (as demonstrated here) or tools available elsewhere^(^^28^^,^^36^^)^ and then loaded in napari. In our experience, simple thresholding methods to label either charging centers or full artifacts do not work well due to the presence of other features of similar intensity. Correct annotation of the charging centers is crucial as it provides both an inverted mask of charging locations for correction and an indication of where the charging artifact is relative to the charging center, hence being useful for choosing initial values during optimization of the functions used (see below).

The filtering scheme works in the following way. First, it takes the previous rows (Figure 4a, red box) and averages perpendicular to the scanning direction (vertical here). This is used as an approximation of the signal without the charging effect. The difference between the current row (Figure 4a, blue arrows) and the previous-row average is assumed to be an estimation of the effect of the charging (Figure 4c–e, blue line). Simply subtracting this estimation of charging effects from the current row of data gives noisy results from row-to-row. Instead, the tails of the charging effect signal (artifact that is outside of the charging center) can smoothed or modeled by curves, and when subtracting this from the original signal, it gives results that are more consistent from scanning row to row. Throughout this data processing tasks, the data within the charging artifact region is not considered in the calculations; the charging artifact label in Figure 4b is used as a negative mask and also to direct the direction of the function to optimize in either side (see below).

We have trialed several functions that may fit and optimize best to the charging artifact tail signals. Exponential ( 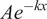 ) and Gaussian functions ( 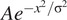 ) can fit reasonably well to these tails but overall give poor results when reconstructing images, in particular at the function optimization step the Gaussian filter often fails to converge to suitable parameters (see Supplementary Figure S5). Some of the reasons why it fails to converge is that the signal is often flat in the rows above the charging artifacts, while other times, there are other biological features nearby that interfere with the function fitting, leading to unrealistic curve fitting parameters. Instead, we found that a shifted logistic sigmoid function (or Fermi-Dirac distribution type) works very well for this task. This function appears like a smoothed step function and is widely used in machine learning algorithms as an activation function, having the advantage that it “saturates” on either side of the curve, which is more characteristic of the charging artifact tails observed. Because the tails themselves were asymmetrical, the functions used to mitigate them were different for the left side and right side of the charging artifacts, and given by:(3)

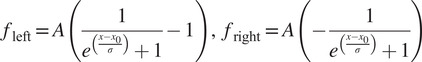

with 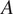 , 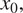 and 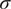 being parameters to be fitted, and functions 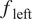 fitted on the left of the labeled artifact, and 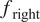 fitted on the right side.

After optimizing 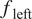 and 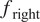 (Figure 4d, red and purple curves, respectively) these functions are subtracted from the row signal (excluding masked regions), resulting in a charge-mitigated signal that still represents the charging object (Figure 4e, brown). Running this process row-by-row in up-down and down-up passes results in a filtered image (Figure 4f), which is a significant improvement compared to Figure 4a, suppressing the main artifacts, while recovering some of the washed-out data previously hidden below.

In addition to SEM images of lipid droplets found in yeast cells, a second dataset featuring myelinated sheaths from mouse brains was used to test this process (Figures 4g–i). The manual segmentation of charging centers (Figure 4h) included both the charging centers and whole organelles, despite many regions not displaying charging artifact tails (including these noncharging regions has no negative impact, see Supplementary Figure S7). The filtered image (Figure 4i) demonstrates a substantial visual improvement to the image quality. We note that in regions near significant charging effects, although the contrast can be matched to the remaining image, there are no recoverable biological features present. This is particularly noticeable around the elongated diagonal biological feature on the right side of the image.

The main advantage of mitigating charging artifacts is the improved contrast of organelles near the charge centers. As such, both manual and automatic segmentation tools are expected to perform better when charging artifacts are absent, as these are notoriously sensitive to contrast changes which could lead to poor visualization and missing or misidentified organelles.

To better understand the effects of our charge mitigation scheme, we have compared the background in the vicinity of charging centers in corrected images (e.g., Figure 4f or i) to their uncorrected counterparts (e.g., Figure 4a or g), taking care to exclude regions that have been labeled as charging centers from the calculation. Through filtering, we expect to “uncover” biological features that were previously obscured by the charging tails and as such would expect a decrease in the standard deviation of the signal intensity around the charging center. The standard deviation of the signal intensity of the results shown in Figure 4f is 60% lower than the signal intensity in Figure 4a. Similarly, the standard deviation signal in Figure 4i (excluding labeled regions) is 58% less than in Figure 4g, suggesting the regions previously obscured by the charging artifacts are now in a more comparable range of signal intensity to the regions throughout the rest of the dataset. Through visual inspection, it is clear that while some information is recovered, at close proximity to the charging centers information is lost due to the presence of the charging artifact.

## 5. Resolution Estimation Using One-Image FRC

Measuring image quality enables users to evaluate the best imaging protocols for the requirements of their research question. Image resolution is a helpful measure of image quality as it can be directly related to physical dimensions, so it is intuitive to interpret. Resolution can be defined as “a maximum spatial frequency at which the information content can be considered reliable”^(^^37^^)^.

One method of determining image resolution is via a two-image Fourier Ring Correlation (FRC)^(^^38^^)^, which has been implemented in fluorescence microscopy. It is both a measure of resolution and consistency between images within a dataset. In this method, two independently acquired images of the same field-of-view are compared in the frequency domain to find the highest frequency where the images can be said to be similar. This is performed by determining the frequency where cross-correlation drops below a given threshold, often 1/7 = 0.143, as commonly employed in cryo-electron microscopy^(^^39^^,^^40^^)^. The requirement for two independently acquired images is challenging to fulfill as it cannot be done retrospectively, and repeated acquisition could introduce image artifacts (i.e., beam damage), affecting the resolution.

Koho *et al.* proposed a one-image FRC calculation based on subsampling a single image to produce pairs of images^(^^41^^)^. This method arranges alternate pixels of an image in a checkerboard pattern to create subimages from a subset of pixels and then calculates the FRC between the subimages. However, in reality, the calculated FRC from these subimages is not equal to the value determined from two separate experimental images, but it can be calibrated (see below). A calibration function is then applied to match the one-image FRC from subimages to the gold-standard two-image FRC for the specific microscope and imaging conditions used.

The method of Koho *et al.* has been adapted for serial FIB/SEM imaging in a software tool called Quoll^(^^42^^)^. Quoll is an open-source, user-friendly tool, and library to calculate the local resolution of single images with the one-image FRC. This method can be applied to any imaging modality once the calibration has been performed. The 2D image is first split into tiles, and the one-image FRC is calculated on each, returning a map of the spatial variation of resolution across the image, and a plot of its distribution. This process can be done on multiple 2D images within a 3D stack to understand resolution throughout the dataset. If large regions of featureless background or artifacts are present, it could skew the output as measurements would be taken on areas without appropriate levels of information present; these can be excluded through masking prior to assessment.

### 5.1 Calibration

The FRC curve obtained from the subsampling method of the one-image FRC is shifted from the gold-standard two-image FRC, where the same details in the one-image FRC curve are shifted to lower frequencies than they should be. As a result, resolution values reported by the one-image FRC are higher than the gold-standard two-image FRC, so calibration of the one-image FRC is required to match the resolution obtained by both methods. The rationale for this calibration is explained in further detail by Koho *et al.* in their original implementation of the one-image FRC resolution measurement^(^^41^^)^. This is an instrument-specific calibration as it is affected by the noise generation of the instrument. The calibration dataset requires two repeated images of the same field of view taken at several pixel sizes/magnifications. Ideally, there should be no significant, artificial changes between the images such as artifacts or image shifts, as these could affect the two-image FRC that is calculated between them. If necessary, image registration can be used to correct shifts and choosing specimens which do not suffer from specimen degradation through repeated imaging is helpful (i.e., inorganic options such as gold or polystyrene beads).

**Figure 5.**
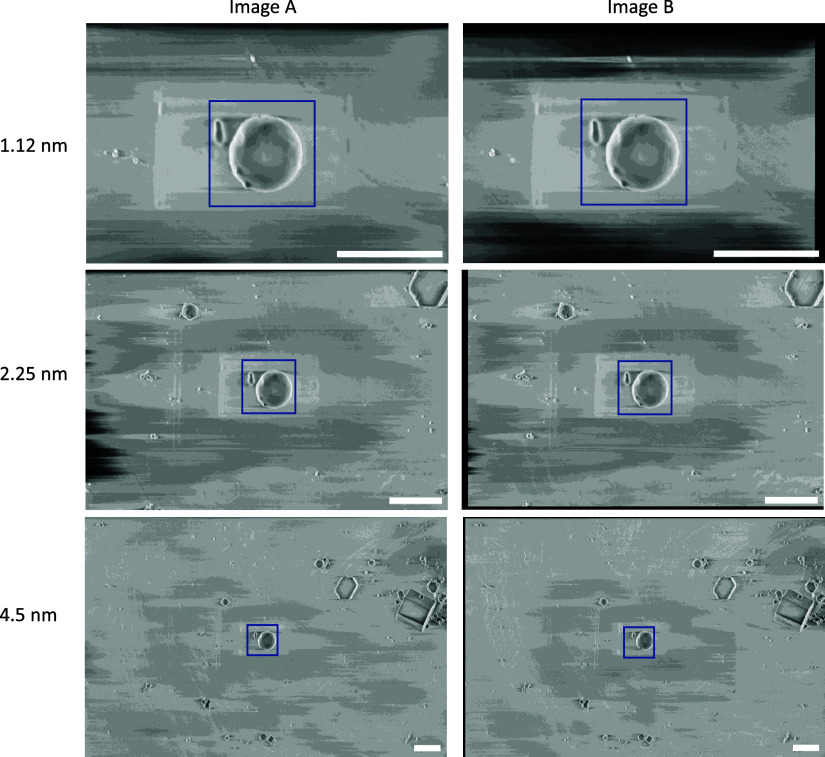
Calibration dataset of polystyrene beads used to calculate the gold-standard two-image FRC. These images were taken at 90° SEM angle. A region-of-interest (blue box) covering 2 × 2 μm^2^ was used for the calibration, where the average one-image FRC and two-image FRC were calculated for these regions at all pixel sizes. Scale bars represent 2 μm.

In our recent cryogenic pFIB/SEM study^(^^1^^)^, calibration was performed using images of polystyrene beads of 1 μm diameter (Abvigen, Newark, NJ) in cryogenic conditions. The images show the same field-of-view at 1.12, 2.25, and 4.5 nm pixel size. The final dataset consisted of six images, with a pair of images taken at each pixel size and SEM angle (angle between the SEM and the specimen face). The linear stack alignment with SIFT plugin in Fiji 2.3.0/1.53q was used to register each pair of images to each other, to ensure the exact same field-of-view was considered for both images^(^^43^^)^. The images were cropped to cover the same physical field-of-view for all pixel sizes, 2000 × 2000 nm^2^ for the 52° images and 1600 × 1600 nm^2^ for the 90° images (Figure 5). The number of pixels in each pair of images was different due to the varying pixel size.

**Figure 6.**
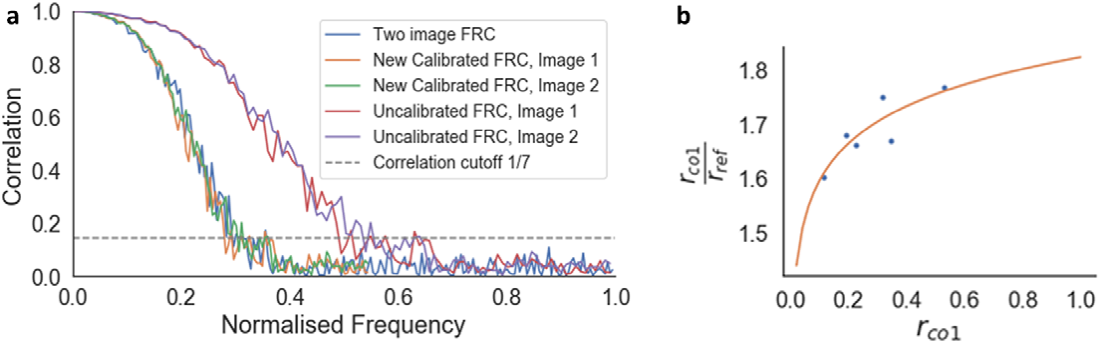
Calibration of the one-image FRC measurement to the gold-standard two-image FRC. (a) Two-image FRC curve and one-image FRC curves before and after calibration for the image pair at 4.5 nm pixel size at 52° SEM angle. (b) 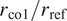 versus 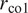 scatter plot (blue dots) and calibration curve 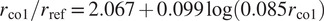 , where 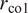 is the average resolution measured from the one-image FRC for the image pair (Figure a, normalized frequency value at the location the curve crosses the correlation cutoff line of 1/7), and 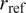 is the resolution from the gold-standard two-image FRC measured from the image pair. Calibration shifted the uncalibrated one-image FRC curves along the frequency axis to match the two-image FRC curves, ensuring that the resolution measurement for the one-image FRC matches the gold standard.

The FRC curve (cross-correlation vs. frequency) was calculated from each pair of images, and normalized to a scale of 0–1, where 1 was the maximum frequency calculated (Figure 6a). This normalization enabled direct comparison of FRC curves between images. The normalized frequency at which the cross-correlation fell below 0.143^(^^39^^)^ was taken as the reference resolution ( 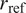 ). The one-image FRC resolution was also calculated following the checkerboard sampling method of Koho *et al.*, this was the 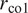 .

A calibration function was fitted to the plot of 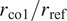 against 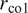 (Figure 6b). This calibration function shifted the one-image FRC curve to match the two-image FRC, so that the resolution from both methods were comparable. This calibration function is instrument-specific, so it can be reused for any images taken from that instrument, provided that the image acquisition parameters are within the range of the calibration dataset.

### 5.2 Application to cryogenic serial pFIB/SEM data

Quoll was used in a recent publication to develop cryogenic serial pFIB/SEM imaging of biological specimens^(^^1^^)^. Here, Quoll resolution measurement was demonstrated on five different biological specimens (*R. rubrum*, HeLa cells, Vero cells, mouse brain, *Saccharomyces cerevisiae*). The measurements were used to show that imaging at 90° SEM angle produced better results than 52°, to determine the depth of field of the instrument, and to show that there was no degradation of image quality through serial sectioning of the specimen, even at depths of ≈25 μm. These performance evaluations would not have been as accurate and exhaustive without the quantitative measurements from image resolution estimation with Quoll.

**Figure 7.**
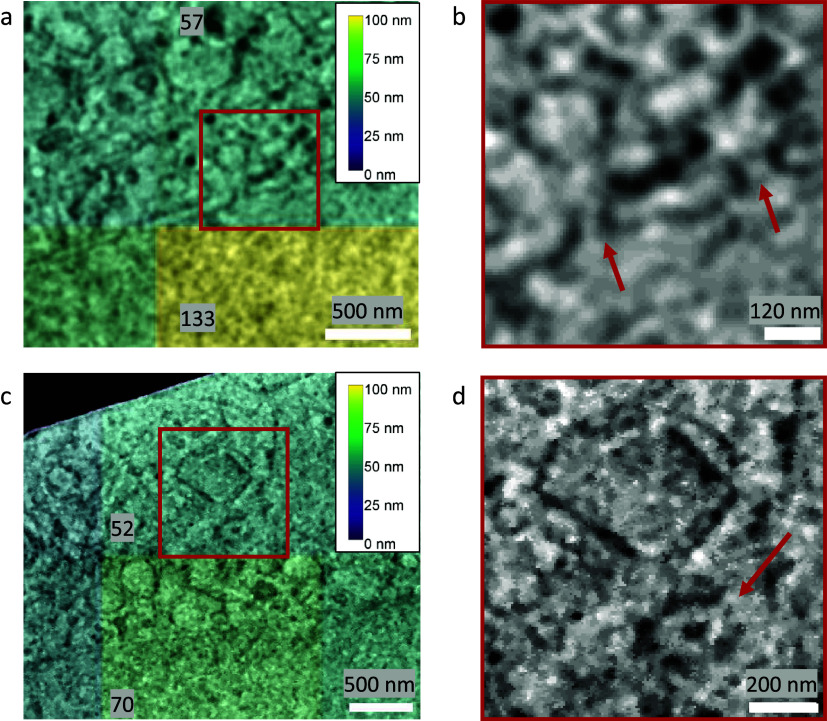
Validation of Quoll resolution measurements compared to physical features of known sizes in HeLa cells. Images were taken with 6.745 nm pixel size at 52° SEM angle. Nuclear pore complexes (a,b) of 120 nm diameter were clearly resolved in the images, as indicated by the arrows in (b), and the resolution in this region was estimated as better than the resolvable features. Similarly, in (c) and (d), centrosomes of 200 nm diameter could be resolved from the images (indicated by the arrow in d), and resolution was better than the size of the centrosomes. (a,c) A resolution heatmap is overlaid onto regions of the image showing nuclear pore complexes and centrosomes respectively, where the colors represent the resolution values of that local region and numbers are the local resolution values in nm calculated on the respective rectangular regions. (b,d) are the zoomed-in regions indicated in (a) and (c) with a red square, with arrows indicating the position of the nuclear pore complexes and centrosomes, respectively. Data is EMPIAR-11419.

The image resolution reported by Quoll was validated against biological structures with known physical sizes. The local image resolution was calculated for a cryogenic serial pFIB/SEM image of HeLa cells on tiles measuring 256 × 256 pixels, which corresponded to a physical field-of-view of 1.73 × 1.73 μm^2^. These images contained nuclear pore complexes and centrosomes, which are approximately 120 and 200 nm in diameter, respectively^(^^44^^,^^45^^)^. These structures were clearly resolvable in the images, and resolution in the tiles containing these structures surpassed the known diameters of these structures, validating the resolution measurements (Figure 7). FRC measurements are carried out on tiles within image slices and the reported measures provide a plot of the mean and standard deviation of resolution across the slice, as well as a heatmap for visualization. It is important to emphasize that these values are not the highest resolution visible within the slice, but instead a representation of the mean resolution of each tile. The goal of this method is to provide a quality metric for the raw data as a whole.

We found that tile size does not affect the overall resolution distribution of the image. The local image resolution was calculated on cryogenic pFIB/SEM images of *S. cerevisiae* and *R. rubrum*, at 3.37 and 1.94 nm pixel sizes, respectively. The images were sampled with tile sizes of 128 × 128, 256 × 256, and 512 × 512 pixels. The difference in minimum and maximum median resolutions for all tile sizes was within 0.38–0.48 pixels. The Kruskal–Wallis H-test was applied with the null hypothesis that the population median of all groups was equal^(^^46^^,^^47^^)^. The null hypothesis could not be rejected (*p* > 0.05) so the median resolution for all three tile sizes was considered equal (Table 2).

**Table 2.** Summary statistics of resolution distribution measured from different tile sizes.

Tile size	Number of tiles	Mean resolution (nm)	Standard deviation of resolution (nm)	Median resolution (nm)
*S. cerevisiae,* pixel size 3.3724 nm				
128	256	45.2	9.68	45.3
256	64	47.5	10.4	46.6
512	16	44.0	6.96	45.3
*R. rubrum,* pixel size 1.94171 nm				
128	64	20.9	5.31	19.7
256	16	21.1	5.31	20.6
512	4	20.3	1.67	20.3

*Note.* Tile size was not found to affect the overall resolution distribution of the image. The resolution distribution median was found to be equal for all tile sizes (*p* > .05) by the Kruskal–Wallis H-test. All measurements are rounded to three significant figures.

## 6. Discussion

Cryogenic serial pFIB/SEM imaging provides exciting opportunities for *in situ* structural biology, though as a relatively new method of imaging, requires development of appropriate computational tools both for assessment of the method and processing of the data to enable biologically relevant qualitative and quantitative outcomes. Okapi-EM has been developed to begin this process. It includes three plugins to align serial SEM stacks, mitigate charging artifacts and to assess the resolution of SEM data. In future, Okapi-EM will also include a separate plugin to measure and mitigate curtaining artifacts.

Many of the approaches developed here for cryogenic serial pFIB/SEM are likely also applicable to nonplasma-based and/or room temperature SBF/SEM or FIB/SEM or serial TEM (serial section TEM or array tomography) with little or no modifications needed to the method or implementation. If modifications are needed, we are happy to adapt Okapi-EM to meet these needs and encourage feedback from users and developers through contacting the corresponding author here or via our GitHub page.

Okapi-EM will continue to be developed into a more automated, quantitative workflow for data processing. For serial FIB/SEM, alignment of 3D stacks is generally one of the first data processing steps and its outcome can have an impact on all downstream processing and analysis. It is therefore important to apply the minimum amount of transformations (i.e., change the data as little as possible), but do enough to ensure the data is interpretable. Finding this balance is currently a manual process. In the future, development of image alignment quality metrics will enable this to shift to an automated process.

Currently, the next step in data processing for serial FIB/SEM data is mitigation of artifacts such as charging. As described above, our implementation relies on the prior identification of charging centers through either manual or machine learning-based segmentation approaches. Our focus in this area will be on the development of a more automated approach to charge center identification and segmentation. Additionally, we would like to extend our charge mitigation approach to data which display charging in visually different ways. We are aware of charging which is characterized by artifact tails that are bright white surrounding black charging centers or a mixture of white and black charging artifact tails again surrounding black charging centers. Further testing of the filters suggested here, and others are necessary to identify or adapt mitigation strategies for these data types. However, this testing has been hindered by lack of access to publicly archived example data (e.g., in EMPIAR).

Finally, once the data has been aligned and artifacts mitigated, the next steps are visualization, assessment, and segmentation. The FRC resolution measurement is a first step toward quantitative assessment of the output data though others related to “segmentability” (i.e., contrast, information content, presence/absence of a feature of interest) could be developed.

It would also be valid to use image quality assessments at the beginning of the process—data collection—guiding users in optimizing their imaging protocols, where acquisition settings can be determined based on the specified quality metrics and the researcher’s specific question(s). This is especially helpful for new users who may not have the experience to quickly determine the optimum combination of acquisition settings, so image resolution and other quality measurements at the microscope could guide the user to quantitatively optimize their protocols, removing some of the superstitious/anecdotal approaches that are sometimes found in research.

This thought process can then be extended to automated microscopy methods, where acquisition settings (such as electron beam energy) are adjusted during serial imaging to obtain good quality images throughout the session even as the sample features change (e.g., automatically decreasing charging or curtaining artifacts as they appear). This would enable higher quality, longer image acquisition sessions as the user is not required to manually adjust the imaging settings throughout the session. Real-time measurements and parallelized computational approaches will be required for these assessments to quickly identify and react to issues during imaging.

Image quality information can also be saved as metadata with each acquired image or stack of images, which is useful for indexing the images for future reuse. For example, future users could search for images of specific specimen types with a minimum resolution to answer their research question, or methods developers could search for images with low resolution to explore how their methods could improve existing images.

## 7. Conclusion

Volume EM (room temperature or cryogenic) provides exciting opportunities for visualization and quantification of cellular and tissue components, though in many cases, the outcomes of these studies are hampered by the manual nature of data processing and analysis. We have provided a bundle of plugins within the napari data visualization package to speed up the process and ease the burden on researchers using serial SEM or TEM imaging techniques. These tools allow for the alignment of 3D stacks without the introduction of unnecessary transformations, the mitigation of charging artifacts caused during scanning imaging techniques and the assessment of data quality through one-image resolution measurement. These tools are in regular use and development, and we welcome feedback and contributions as they are extended to new data types, imaging modalities, and purposes.

## Data Availability

Cryo-serial-FIB/SEM sample data used for alignment and filtering is available on EMPIAR (https://www.ebi.ac.uk/empiar/) alongside Dumoux *et al*. ^(^^1^^)^. *Saccharomyces cerevisiae* (yeast) data set is EMPIAR-11416, HeLa cells is EMPIAR-11419 and mouse brain is EMPIAR-11415.

## Supplementary material

For supplementary material accompanying this paper visit https://doi.org/10.1017/S2633903X23000119. click here to view supplementary material
