# Supplementary material for: Okapi-EM: A napari plugin for processing and analyzing cryogenic serial focused ion beam/scanning electron microscopy images
Source: Biol Imaging. 2023 Mar 27;3:e9. doi: 10.1017/S2633903X23000119 (PMC10936406; doi:10.1017/S2633903X23000119)
Supplement: Supplementary file 1 [file blgsup.zip › S2633903X23000119sup001.docx]

# Supporting Information


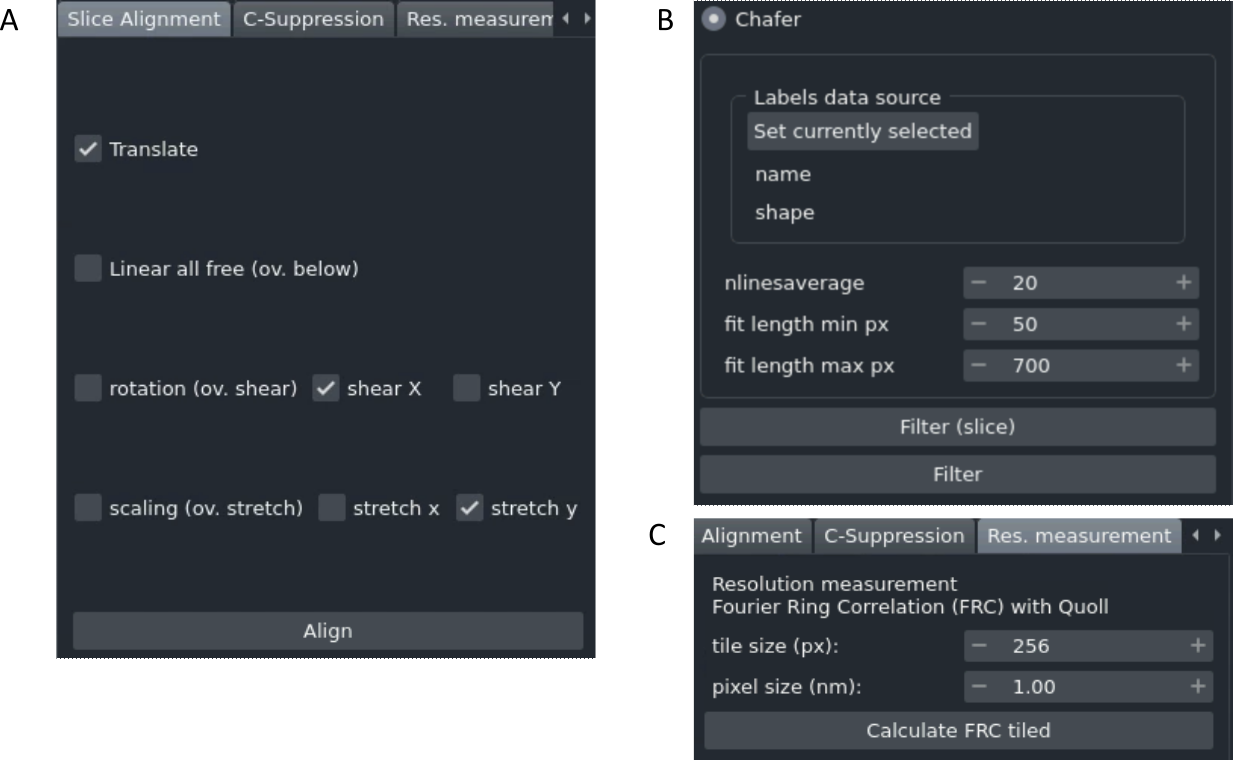


Figure S1. The graphical user interfaces for all three Okapi-EM plugins. (A) In the slice alignment user interface, the user can choose which parameters in the transformation(s) are allowed to be optimized. Defaults are freedom to translate, shear x, and stretch y, which are best for SEM slice stacks. (B) In the charge artifact removal filter interface (chafer) the user can select the labelled charge centres and specify parameters related to the filters. (C) In the resolution measurement interface (Quoll) the user must specify the tile size (in pixels) and the pixel size (in nanometers). After execution, a new napari layer will appear as a heat map overlay, and a statistical summary of the calculated values will appear in the user interface.

The user interface for the slice alignment tool (Figure S1A) offers several options that determine which and how the parameters in the transformation matrix between image slices are optimised. *Linear all free* (also known as affine in other software packages) means that all parameters in the linear transformation are adjustable during the optimization. If the *rotation* option is selected, rotation is optimized, but shearing along X or Y axis are not (overridden), being equivalent to *rigid body* in other alignment algorithms. If rotation is deselected, shearing along x or y directions can be individually set as adjustable. If *scaling* option is selected, a single scaling value for both horizontal and vertical directions is adjustable during optimization. With this option deselected, the scaling along vertical and horizontal directions can be allowed/disallowed individually.

The user interface for the charge mitigation using chafer is shown Figure S1B. To run this tool, a napari layer associated with the image data is needed, where the charge centres are annotated. This can be set with the button provided. *nlinesaverage*, *fit length min px* and *fit length max px* are adjustable parameters. *nlinesaverage* sets the number of scanning lines to use to calculate background signal before optimising sigmoid curves. A higher value gives a better estimation of the nearby signal intensity without the charge artifact but it will run slower and if the image has many edges along the vertical direction it may perform worse. *fit length min px* sets the minimum data points (pixels) next to the annotated artifact that are needed to attempt filtering. This is a way to avoid filtering if the charge artifact is too close to the image limits or too close to another charge artifact. In both these cases the function optimization may otherwise perform poorly. Similarly, *fit length max px*, sets the maximum number of points to be used, so that if data along the line extends further then it will be ignored. If the FIB sections have a short width (non-milled areas appearing on the side of the images), a lower value may prevent image data beyond the sectioning to be used during the function fitting. If this value is too large, more data will be used during the optimization but it may slow calculation significantly. It is recommended that users try different parameters on single images and evaluate the result (*Filter (slice)* button) before attempting to run the filter on a complete stack (*Filter* button).

The resolution estimation tab is shown in Figure S1C. This tool works on two-dimensional data only if data is volumetric, it will calculate in the visualiser selected layer. The calculation splits the image in square tiles with size in pixels being the adjustable parameter *tile size*. The parameter *pixel size (nm)* is needed to get accurate results in the statistical information resulting from the FRC calculation.

In the Linear Stack Alignment, the unconstrained transform of feature vectors between slices is given by:

$$\left[ \begin{matrix} x' \\ y' \end{matrix} \right]=\left[ \begin{matrix} p_{1} & p_{3} \\ p_{2} & p_{4} \end{matrix} \right]\cdot\left[ \begin{matrix} x \\ y \end{matrix} \right]+ \left[ \begin{matrix} x_{t} \\ y_{t} \end{matrix} \right]=M\cdot\left[ \begin{matrix} x \\ y \end{matrix} \right]+\vec{t}$$

The transformations used when constrains are imposed are summarized in the table below.


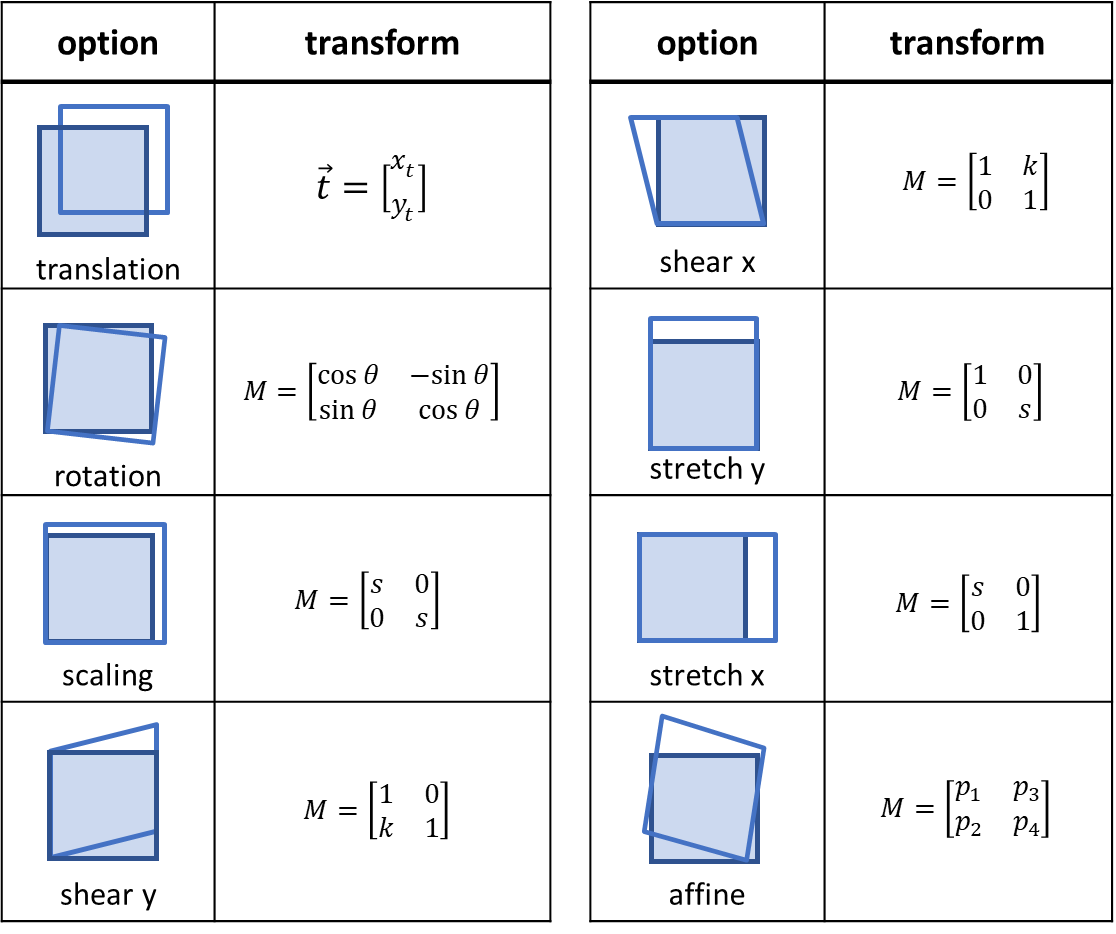


Figure S2. Transformation options available in Okapi-EM as well as their mathematical representations.


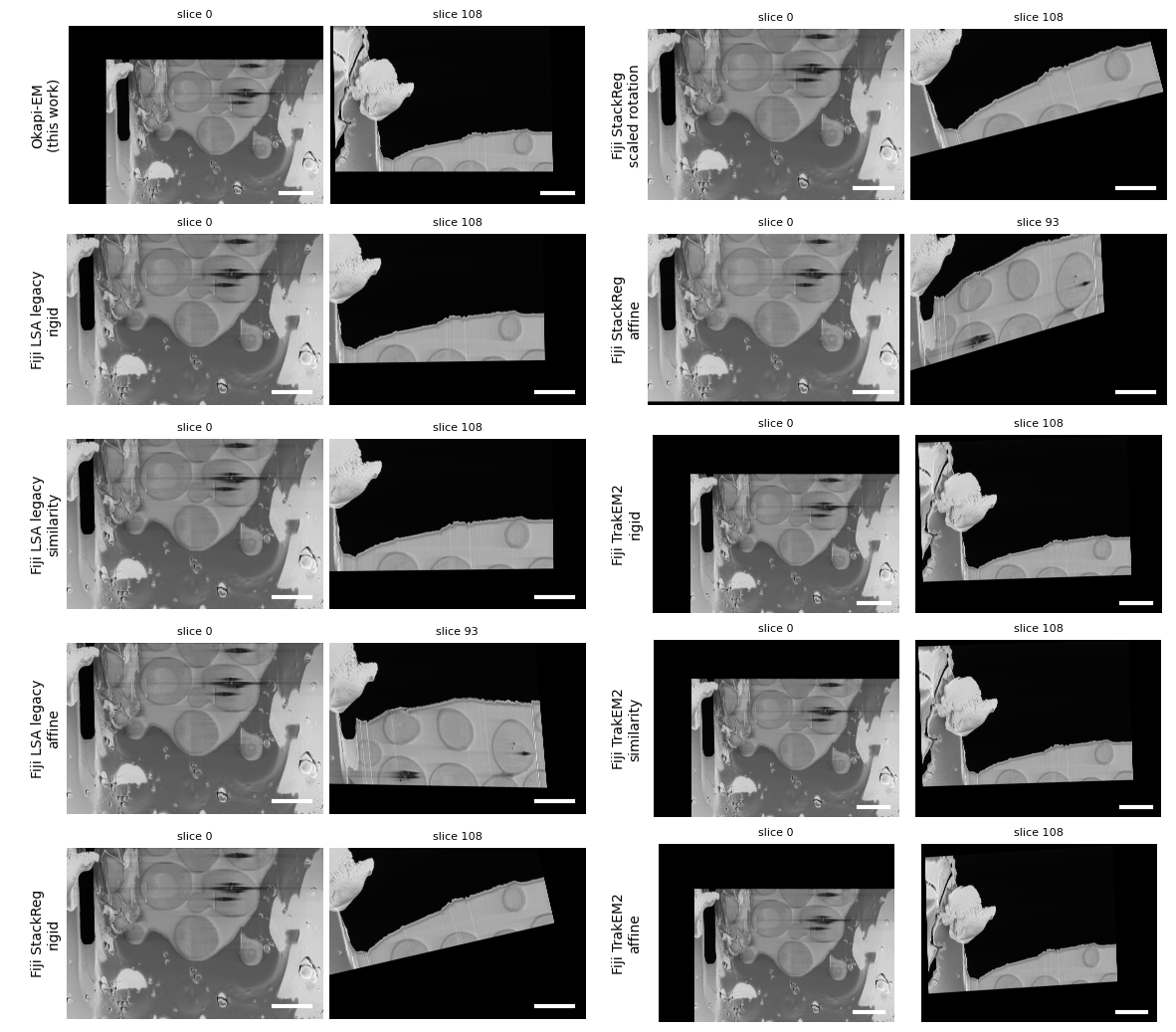


Figure S3. Comparison between different stack alignment algorithms. Various stack alignment algorithms were applied to the same yeast cryogenic serial pFIB/SEM dataset (EMPIAR-11416). The first and last slice of the stack are presented side-by-side as a means to highlight accumulated matrix transforms that result in excessive rotation in images, which may not reflect the reality of the experiment. All scale bars correspond to 3 µm.

Some important observations to note from the stack alignment results above:

- In most non-okapi alignment algorithms the last image slice is rotated compared to the first slice. StackReg alignments generally result in excessive image rotation.
- Some LSA-Fiji alignments crop slices towards the end (rather than expanding XY image dimensions), which can result in data loss.
- Algorithms *Fiji Legacy - affine* and *Fiji StackReg - affine* did not complete alignment of the whole stack due to errors.


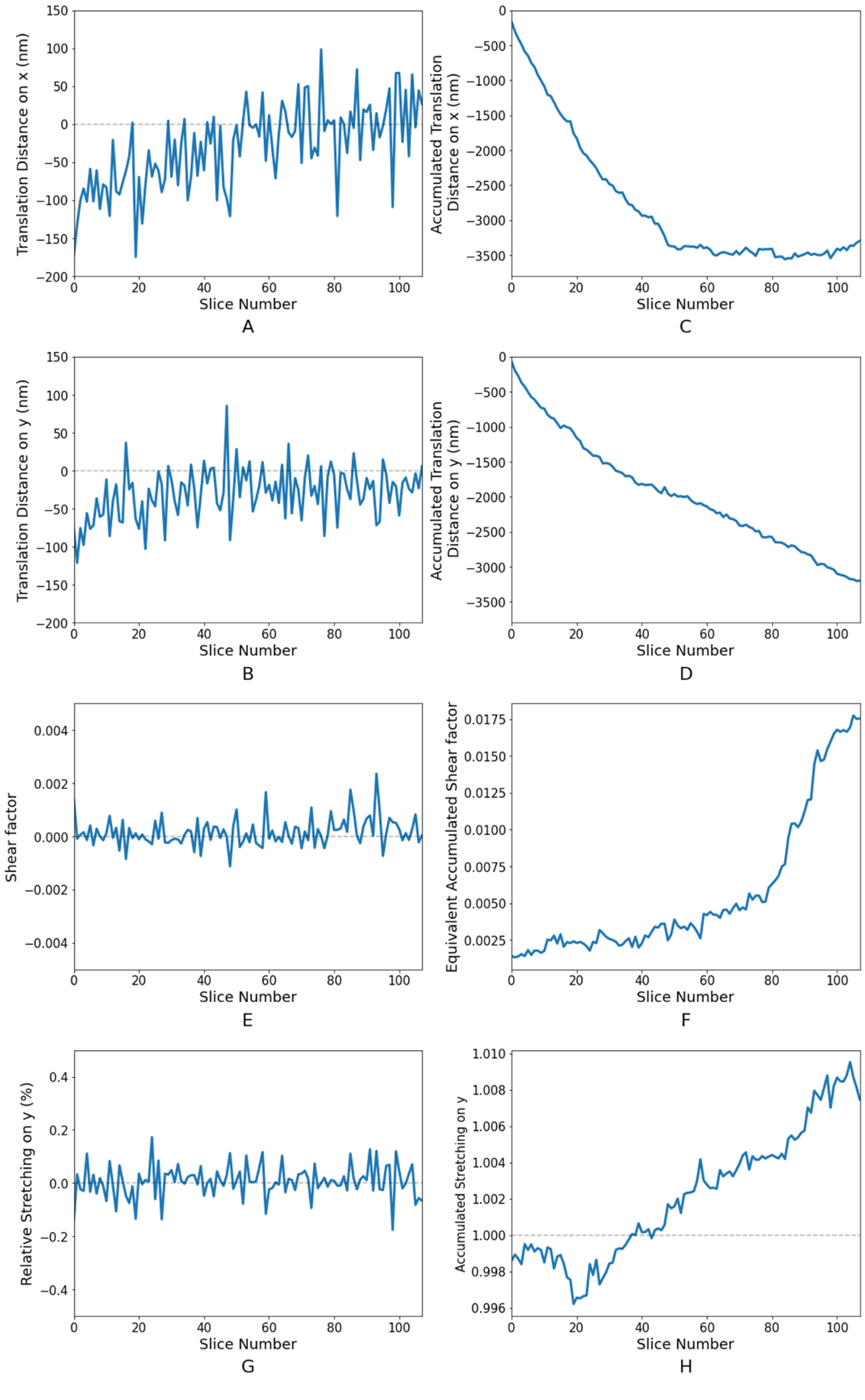


Figure S4. Transformation parameters along the image stack calculated for the yeast data set and using the linear stack alignment included in Okapi-EM, with {translate, shear x, stretch y} selected. (A)-(B) translation distance on x and y directions respectively, (C)-(D) accumulated translation distance on x and y directions respectively, (E) shear-x factor ($k$), (F) equivalent accumulated shear-x factor, calculated from multiplying matrices for all previous image slices, where value presented in this plot is $p_{3}/p_{4}$, (G) relative stretching on y direction, (H) accumulated stretching on y direction.


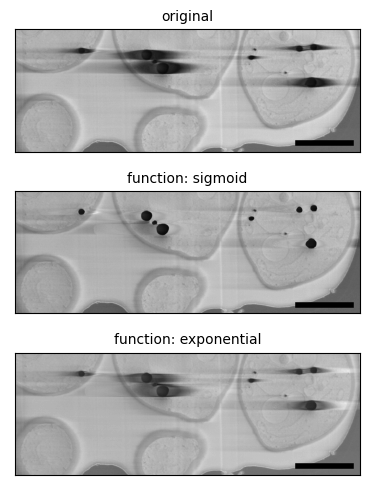


Figure S5. Comparison of results obtained when sigmoid and exponential functions were used for mitigating the charge artifacts using a yeast sample (EMPIAR-11416). In all calculations, the parameters nlinesaverage, fit length min px and fit length max px used were the default 20, 50 and 700, respectively. Scale bars represent 2 µm.

Visually, the sigmoid filtering gives better results when compared with the exponential method. The exponential filter eliminates some of the tails but the suppression of the artifacts is not as extensive as the sigmoid, and some charging artifacts exhibit additional brightening of the tails on the right side. Chafer tool in Okapi-EM uses the sigmoid implementation of the filter. A gaussian function was also tested and it only successful optimizes in *scipy.optimize* with excessive manual adjustments on a per-row basis. Generally, less than 1% of the optimizations succeed in a full image, making it inconvenient to use or implement.

As mentioned in the main text, the standard deviation of signal intensity with the charging artifacts masked can be used as a measure of quality of this filter performance, with lower values indicating better removal of the charge artifact tails. For each of the images in Figure S4, from top to bottom, the values obtained are as follow: 23.8, 18.2, 22.4, with the sigmoid filtering presenting a lower value consistent with the visual observations described here.


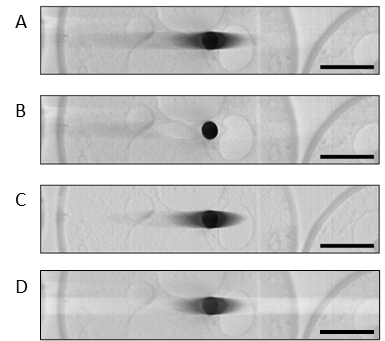


Figure S6. Comparison between charge artifact mitigation filters (A) SEM image of a lipid charge artifact in a yeast sample (EMPIAR-11416); (B) filtered using chafer in Okapi-EM, (C) using the method described in Spehner et al. ^(1)^, and (D) filtered using an FFT bandpass filter in Fiji-ImageJ. All scale bars represent 1 µm.

The charge artifact suppression algorithm presented in Ref (1) by Spehner et al. and further detailed in the “Appendix A. Supplementary data”, was tested with the charging artifacts in our images, as described in the publication. However only minor suppression is observed (Figure S5C), mainly further away from the charging centre.

The FFT bandpass filter in Fiji ImageJ was also tested and it did not result in a substantial charge suppression (Figure S5D). The filter settings used were:

- structures by size: zero
- stripe suppression: horizontal and tolerance of direction 5%
- no autoscaling of intensities.


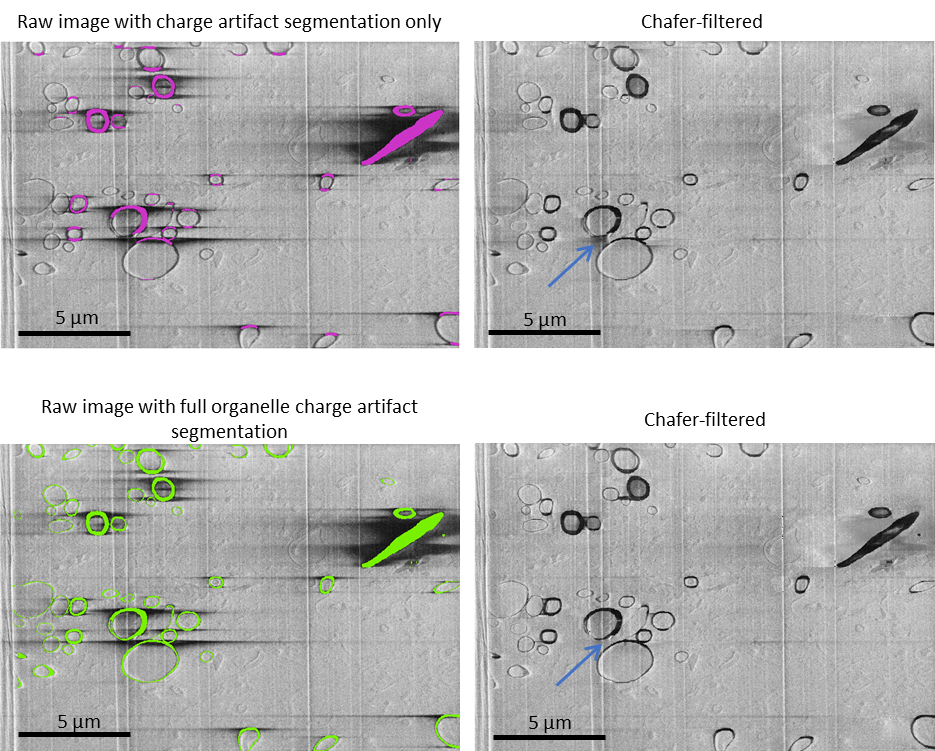


Figure S7. Chafer performance when only charging features are included vs when whole organelles (regardless of charging status) are included. Left, SEM images of mouse brain (EMPIAR-11415). Left, top, where only charging artifacts have been segmented as indicated in pink; in left, bottom the whole organelle responsible for the emergence of charging artifacts (myeline sheaths) is fully segmented in green. Right column is the result after running the chafer filter using the respective labels on the left. The resulting images are visually similar, with the most significant difference indicated with a blue arrow.

| Okapi-EM tool | Execution time |
| --- | --- |
| Linear Stack Alignment | 25 minutes |
| Charge artifact suppression using chafer | 41 minutes |
| Fourier Ring Correlation estimation (single image slice) | 8 seconds |

Table S1. Execution times of major Okapi-EM's tools, used in experimental cryo-FIB/SEM yeast data, applied to the whole image stack, comprised of 109 image slices, each with a resolution of 6144x4096 pixels. Computer was equipped with Intel Xeon Gold 5218 (6 core) CPU, and with 80Gb RAM.


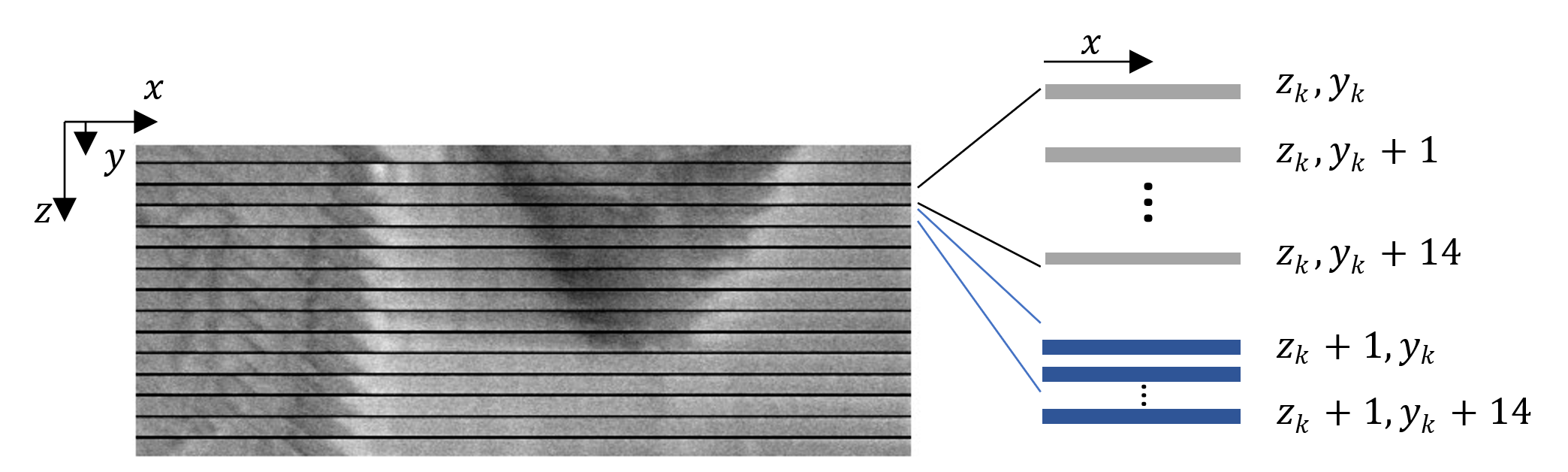


Figure S8. Illustration on how cross-sectional views were rendered for Figure 3 in main text.

Here we describe how the cross-sectional views shown in Figure 3 were rendered. The data presented consists of 109 image slices, and each image has pixel size of 6144 and 4096 along vertical and horizontal direction respectively. A cross-section along the width-stack directions would be an image with size 109 x 6144 pixels that, if presented with square pixel size, the image would too thin to see any detail if presented here. One solution would be to extend the dimension of the pixel size along the vertical direction by about $N$ times and using interpolation, but we found that this does not give satisfactory information and makes evaluation of the alignment difficult. In the rendering presented here, for each row line of the stack at given $z_{k}$ and $y_{k}$ pixel coordinates, $N=14$ additional row lines were rendered below, consisting of the row lines with the same $z_{k}$ and $y_{k}+1$, $y_{k}+2$, …, $y_{k}+14$. We choose 14 additional lines as 15 is how many times the z-slice milling thickness is higher compared with the x and y pixel resolution, hence resulting in microscopic features displaying with nearest 1:1 ratio appearance. This slice-displaying method, although unusual, results in features appearing jagged but it is a way to scale the cross-section image in the vertical direction while at the same time showing additional consecutive data, hence visually aiding evaluation of the alignment. In the figure above, a black horizontal bar was added between the rows only for making clear that it corresponds to different $z_{k}$ pixel coordinates. In the main article caption of Figure 3, these black separating bars are not displayed for aesthetic reasons.

# References

1. Spehner D, Steyer AM, Bertinetti L, *et al.* (2020) Cryo-FIB-SEM as a promising tool for localizing proteins in 3D *Journal of Structural Biology* **211**, 107528.
